# Genome and Transcriptome Sequencing of the Astaxanthin-Producing Green Microalga, *Haematococcus pluvialis*

**DOI:** 10.1093/gbe/evy263

**Published:** 2018-11-29

**Authors:** Qiulan Luo, Chao Bian, Ming Tao, Yu Huang, Yihong Zheng, Yunyun Lv, Jia Li, Chaogang Wang, Xinxin You, Bin Jia, Junmin Xu, Jiancheng Li, Ze Li, Qiong Shi, Zhangli Hu

**Affiliations:** 1 Guangdong Technology Research Center for Marine Algal Bioengineering, Guangdong Key Laboratory of Plant Epigenetic, Shenzhen Key Laboratory of Marine Bioresource & Eco-environmental Sciences, College of Life Sciences and Oceanography, Shenzhen University, Shenzhen, Guangdong, China; 2 Key Laboratory of Optoelectronic Devices and Systems of Ministry of Education and Guangdong Province, College of Optoelectronic Engineering, Shenzhen University, Shenzhen, Guangdong, China; 3 Shenzhen Key Lab of Marine Genomics, Guangdong Provincial Key Lab of Molecular Breeding in Marine Economic Animals, BGI Academy of Marine Sciences, BGI Marine, BGI, Shenzhen, Guangdong, China; 4 Centre of Reproduction, Development and Aging, Faculty of Health Sciences, University of Macau, Taipa, Macau, China; 5 BGI Education Center, University of Chinese Academy of Sciences, Shenzhen, Guangdong, China; 6 Shenzhen Engineering Laboratory for Marine Algal Biotechnology, Longhua Innovation Institute for Biotechnology, College of Life Sciences and Oceanography, Shenzhen University, Shenzhen, Guangdong, China; 7 These authors contributed equally to this work

**Keywords:** genome sequencing, assembly, annotation, astaxanthin, *Haematococcus pluvialis*

## Abstract

*Haematococcus pluvialis* is a freshwater species of Chlorophyta, family Haematococcaceae. It is well known for its capacity to synthesize high amounts of astaxanthin, which is a strong antioxidant that has been utilized in aquaculture and cosmetics. To improve astaxanthin yield and to establish genetic resources for *H. pluvialis*, we performed whole-genome sequencing, assembly, and annotation of this green microalga. A total of 83.1 Gb of raw reads were sequenced. After filtering the raw reads, we subsequently generated a draft assembly with a genome size of 669.0 Mb, a scaffold N50 of 288.6 kb, and predicted 18,545 genes. We also established a robust phylogenetic tree from 14 representative algae species. With additional transcriptome data, we revealed some novel potential genes that are involved in the synthesis, accumulation, and regulation of astaxanthin production. In addition, we generated an isoform-level reference transcriptome set of 18,483 transcripts with high confidence. Alternative splicing analysis demonstrated that intron retention is the most frequent mode. In summary, we report the first draft genome of *H. pluvialis*. These genomic resources along with transcriptomic data provide a solid foundation for the discovery of the genetic basis for theoretical and commercial astaxanthin enrichment.

## Introduction

**Fig 1.**
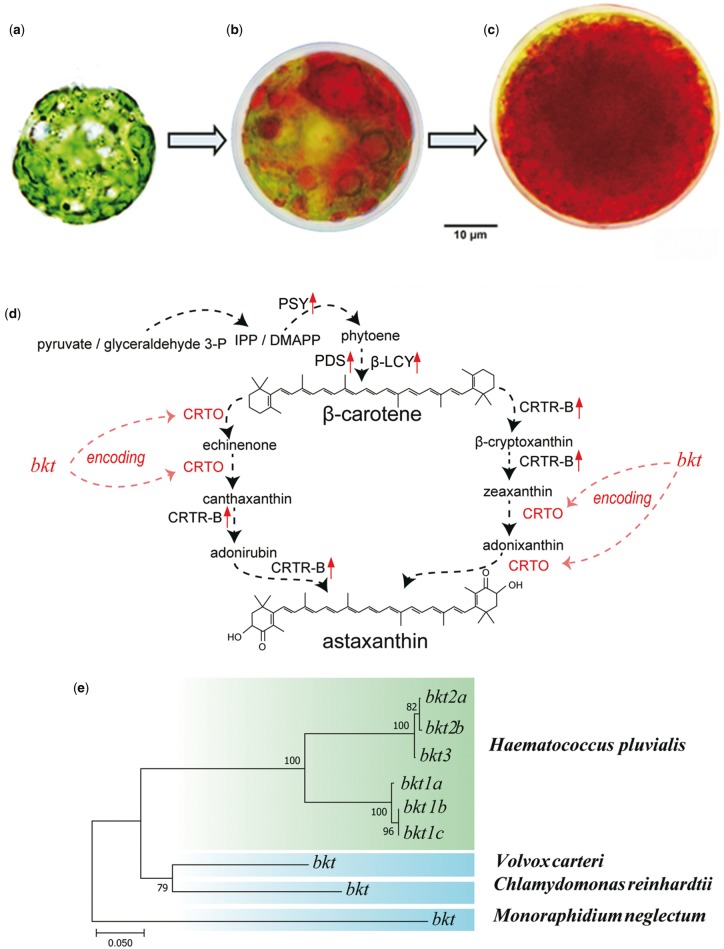
—The life cycle of *Haematococcus pluvialis* and the phylogeny of *bkt* genes for astaxanthin biosynthesis. (*a*) Green motile cell, (*b*) cell under stress, and (*c*) red immobile cell. (*d*) The pathway of astaxanthin biosynthesis (modified from Grunewald et al. 2001) revealed the important roles of CRTO (β-carotene ketolase), which was encoded by the *bkt* genes. (*e*) A total of six *bkt* genes were identified in the genome assembly of *H. pluvialis*, suggesting that multiple gene duplications occurred during genome evolution. In contrast, only a single *bkt* homologous sequence was identified in each closely related species such as *Volvox carteri*, *Chlamydomonas reinhardtii*, and *Monoraphidium neglectum*.

*Haematococcus pluvialis* is a unicellular green alga and is considered as the best natural resource for astaxanthin, which is a high-value carotenoid with strong biological activity for the food, feed, and pharmaceutical industries (Ambati et al. 2014). It has an interesting life cycle with a remarkable division between green motile and red immobile stages (fig. 1*a–c*). It enters the green motile stage under favorable environmental conditions. During their vegetative growth, *H. pluvialis* cells are spherical, ellipsoidal, or pear-shaped with flagella and chloroplasts (fig. 1*a*). When exposed to unfavorable environmental or stress conditions, *H. pluvialis* cells develop into red immobile cells (also called cysts; fig. 1*c*) by losing their flagella, increasing their cell size, forming thick cell walls, and accumulating astaxanthin (Shah et al. 2016).

Transcriptomics-, metabolomics- and proteomics-based studies have revealed proteins involved in astaxanthin biosynthesis under stress conditions, such as high irradiation, nitrogen deprivation, or nutrient starvation (Kim et al. 2011; Su et al. 2014; Gao et al. 2015). However, because of limited genome information, how *H. pluvialis* regulates astaxanthin biosynthesis at the DNA level remains unclear. Meanwhile, these genomic resources will help to breed novel strains of *H. pluvialis* that could have higher astaxanthin yield. We were thus prompted to perform whole-genome sequencing, assembly, and annotation of this economically important microalga. In addition, carotene biosynthetic genes cooperate with β-carotene ketolase (CRTO) and hydroxylase (CRTR-B) to synthesize astaxanthin (fig. 1*d*) under high irradiation and salinity, which are the most common stresses that occur during *H. pluvialis* cultivation (Boussiba and Vonshak 1991). We therefore performed additional transcriptome sequencing on stressed cells to reveal additional genes that are potentially involved in the synthesis, accumulation, and regulation of astaxanthin production.

## Materials and Methods

### Sample Materials, Genomic DNA Extraction, and Genome Assembly

*Haematococcus* *pluvialis* 192.80 was purchased from the SAG Culture Collection of Algae (Göttingen, Germany). The alga cells were cultivated in ESP Ag medium as we reported previously (Zheng et al. 2017; see more details in the following section on Total RNA Isolation), and genomic DNA was isolated from cultured cells using Qiagen GenomicTip100 (Qiagen, Germantown, MD, USA). We applied the traditional whole-genome shotgun sequencing strategy (Lin et al. 2016) and built seven diverse paired-end libraries, including three short-insert libraries (250, 500, and 800 bp) and four long-insert libraries (2, 5, 10, and 20 kb). About 83.1 Gb of raw reads were generated from the seven libraries using the Illumina HiSeq 2500 platform (Illumina, San Diego, CA, USA). After removing the low-quality (containing 10 or more Ns and low-quality bases with quality scores ≤7) and redundant reads, we obtained about 60.6 Gb of clean data for further de novo assembling. In addition, the clean reads from the 500- and 800-bp libraries were employed in the estimation of the genome size of *H. pluvialis* (see detailed methods in Li et al. 2010), which was about 935.3 Mb.

To assemble the whole-genome sequence, we employed the SOAP-denovo2 software (Luo et al. 2015) (with -k 65) to build contigs and primary scaffolds by utilizing reads from the short-insert libraries (250, 500, and 800 bp). Subsequently, reads from the long-insert libraries (2, 5, 10, and 20 kb) were mapped onto the contigs to shape corresponding scaffolds. The Gapcloser (in the package of SOAP-denovo2) was employed to fill the gaps in the scaffolds.

### Genome Annotation

Before annotating gene structures of the *H. pluvialis* genome, we identified repeat sequences using multiple programs including Tandem Repeats Finder (Benson 1999), LTR_FINDER (Xu and Wang 2007), RepeatProteinMask, and RepeatMasker (Chen 2004). Tandem Repeats Finder was employed to search for tandem repeats in our genome assembly using the following parameters: Match = 2, Mismatch = 7, Delta = 7, PM = 80, PI = 10, Minscore = 50, and MaxPerid = 2,000. A de novo repeat library was built by the LTR_FINDER (version 1.0.6; parameter: -w 2). Subsequently, the RepeatMasker was utilized to align our genome sequences onto the Repbase TE (version 3.2.9; Jurka et al. 2005) to search the known repeat sequences as well as map onto the de novo repeat libraries to identify novel types of repeat sequences.

We then performed annotation of the *H. pluvialis* genome assembly with three approaches, including homology-based, transcriptome-based, and ab initio annotation. We selected several representative species, including *Paramecium tetraurelia* (Aury et al. 2006), *Saccharomyces cerevisiae* (Kellis et al. 2004), *Symbiodinium kawagutii* and *Symbiodinium minutum* (Lin et al. 2015), *Chlamydomonas eustigma* (Hirooka et al. 2017), *Chromochloris zofingiensis* (Roth et al. 2017), and *Micromonas pusilla* (Worden et al. 2009) to perform the homology annotation. The protein sequences from abovementioned species were aligned onto our genome sequences utilizing the TblastN (Mount 2007) with E-value ≤ 1e−5. Genewise 2.2.0 (Birney et al. 2004) was subsequently employed to predict possible gene structures based on all TblastN results. Total RNA was extracted from control cells (sample ID: LLMT4, 5, and 6; see more details in the following section on Total RNA Isolation) for subsequent transcriptome sequencing using an Illumina HiSeq 4000 platform. We utilized Cufflinks (version 2.2.1; Trapnell et al. 2010) to identify the preliminary genes. Moreover, Augustus (Stanke et al. 2006) and Genscan (Cai et al. 2014) were selected for ab initio annotation using the repeat-masked genome sequences. Finally, we employed GLEAN software (Elsik et al. 2007) to integrate all genes predicted from the three annotation procedures.

### Total RNA Isolation and Transcriptome Assembly

*Haematococcus pluvialis* cells were cultured in 250 ml Erlenmeyer flasks with ESP Ag medium, statically incubated at 22 °C under a light intensity of 25 μM photons/m^2^/s with a 12-h light/12-h dark cycle (Zheng et al. 2017). When in the logarithmic phase, these cells were mixed and divided into six groups. Triplicated groups of HLST (HLSTA, HLSTB, and HLSTC) were treated with high irradiation (550 μM photons/m^2^/s) and high salinity (45 mM of sodium acetate, as referred by Su et al. 2014) with a pH value of 7.0 for 1.5 h, whereas triplicated groups of LLMT (LLMT4, LLMT5, and LLMT6) were used as controls (25 μM photons/m^2^/s, ESP Ag medium, pH 7.0). Total RNA from each sample was isolated and the corresponding cDNA library was separately constructed for subsequent sequencing (RNA-seq) on an Illumina HiSeq 4000 platform. Paired-end raw reads were then processed by removal of adapters and low-quality sequences using SOAPnuke software (v. 1.5.6; Chen et al. 2018) with default parameters. The remaining clean data were mapped onto the assembled genome with HISAT (Kim et al. 2015).

Transcript quantification in each sample [fragments per kilobase per million (FPKM); Mortazavi et al. 2008] was realized using RSEM (Li and Dewey 2011). Differentially expressed genes (DEGs) between treatment and control groups were identified using DESeq2 (Love et al. 2014) with log2 (ratio) ≥1 and the adjusted P value P_adj_ ≤ 0.05 as threshold. Finally, pathway enrichment analysis was performed with these up- and down-regulated DEGs according to Kyoto Encyclopedia of Genes and Genomes (KEGG) database (Ogata et al. 1999). We finally generated a total of 309,962,820 high-quality clean reads. The total mapping ratio of each library to the genome assembly ranged from 83.71% to 85.65%, and the number of transcribed genes in each sample was predicted to range from 22,243 to 22,609 (see more details in supplementary tables S1 and S2, Supplementary Material online). FPKM values of all transcripts in the six samples are provided in supplementary table S3, Supplementary Material online.

### Evolutionary Status of *Haematococcus pluvialis*

To determine the evolutionary position of *H. pluvialis*, we performed a whole-genome phylogenetic analysis on *H. pluvialis* and other 13 related algae, of which complete gene sets or transcriptional data are available. These examined species included four Prasinococcales species (*M.pusilla*, GenBank Accession number: GCF_000151265.2; *Micromonas commode*, GCF_000090985.2; *Ostreococcus tauri*, GCF_000214015.2; and *Ostreococcus lucimarinus*, GCF_000092065.1), four Trebouxiophyceae species (*Chlorella variabilis*, GCF_000147415.1; *Auxenochlorella protothecoides*, GCF_000733215.1; *Coccomyxa subellipsoidea*, GCF_000258705.1 and *Ettlia oleoabundans*, GEEU00000000.1), and five Chlamydomonadales species (*Volvox carteri*, GCF_000143455.1; *Chlamydomonas reinhardtii*, GCF_000002595.1; *Monoraphidium neglectum*, GCF_000611645.1; *Chlamydomonas eustigma*, BEGY00000000.1; and *Oophila amblystomatis*, GFMX00000000.1). The whole-genome gene sets from *H. pluvialis* and other data were aligned by BLAST (version 2.2.6; Mount 2007) to check their homology and to generate a sequence similarity matrix. OrthoMCL was used to distinguish gene families from the sequence similarity matrix, and Markov Chain Clustering (MCL) with default parameters was applied.

We identified single-copy orthologues among all the target species, and these orthologues were aligned with MUSCLE version 3.7 (Edgar 2004). All alignments were combined to form a super alignment file. We first applied the maximum likelihood (ML) method to estimate the phylogenetic topology, which was implemented in PhyML version 3.0 (Guindon et al. 2010). To confirm the topology from ML, we also unutilized Bayesian inference (BI) to estimate the phylogenetic tree again, which was performed in MrBayes (version 3.2.2; Ronquist et al. 2012).

### Identification of *bkt* genes in *H. pluvialis* Genome Assembly and Other Species

The previously identified three BKT protein sequences (BKT1: CAA60478.1, BKT2: BAA08300.1, and BKT3: AAT35555.1) were downloaded from the NCBI. We utilized BLAST version 2.2.6 (Mount 2007) to search the regions of *bkt* genes and their encoding sequences were further predicted by Exonerate version 2.2.0 (Slater and Birney 2005). All the possible *bkt* copies were translated to proteins and aligned together using MEGA version 7 (Kumar et al. 2016) with the Muscle module. We then classified *bkt* genes based on sequence similarity. Furthermore, the three BKT proteins were employed to predict the corresponding *bkt* orthologs in the three phylogenetically closed species including *V. carteri*, *C. reinhardtii*, and *M. neglectum*, which were identified by the phylogenetic analysis. Finally, we interpreted and aligned all extracted encoding sequences from *H. pluvialis, V. carteri*, *C. reinhardtii*, and *M. neglectum* and constructed a phylogenetic topology using the ML method, with 1,000 replicates implemented by PhyML to obtain corresponding node supports.

### Long-Read Sequencing of a Mixed Transcriptome Sample

As earlier described, *H. pluvialis* cells in the logarithmic phase with or without the 1.5-h treatment and about 10^6^ cells from both groups were collected through centrifugation. Total RNA was extracted using the RNA fast 200 kit (Fastagen, Shanghai, China). Only those RNAs with high RNA integrity numbers (RIN > 9.0) were used for subsequent cDNA preparation. Long-read sequencing by PacBio Isoform (Pacific Biosciences, Menlo Park, CA, USA) was performed on the mixture of all the cDNA samples to obtain full-length transcripts without the uncertainty of assembly from short reads.

## Results and Discussion

### Genome Assembly and Annotation

We generated a draft genome assembly with 669.0 Mb in total length and 7,855 scaffolds (>2,000 bp) with a high scaffold N50 of 288.6 kb (table 1). We further utilized the Benchmarking Universal Single-Copy Orthologs (BUSCO) software (Simao et al. 2015) to examine the completeness of our present assembly. The results demonstrated 59% complete and partial eukaryote BUSCO orthologues. It seems that the genome of *H. pluvialis* is very complicated, we therefore added the PacBio transcriptome data to fill up more gene regions. We finally used the de novo assembled transcripts (sequenced by HiSeq) to map the final gene set from both the genome assembly and the transcriptomes, and observed that this set covered 90% of transcripts (supplementary table S1, Supplementary Material online). We further identified and classified repeat sequences, which account for about 32.2% of the assembled genome (table 1).

**Table 1:** Summary of the genome assembly and annotation

Genome assembly	Parameter
Contig N50 size (kb)	8.2
Scaffold N50 size (kb)	288.6
Assembled genome size (Mb)	669.0
Genome coverage (×)	83.1
Longest scaffold (bp)	1,782,609
Genome annotation	
Number of protein-coding genes	18,545
Transposable elements content (%)	32.2

The complete gene set is composed of 18,545 genes, with an average of 8.7 kb in length. All protein sequences of the GLEAN results were mapped onto the public TrEMBL, Swiss-Prot (Bairoch and Apweiler 2000; Boeckmann et al. 2003), InterProScan (Finn et al. 2014), and KEGG (Ogata et al. 1999) databases using BlastP software with E-value ≤ 1e-5. Finally, approximately 75.8% of the predicted genes have at least one related function assignments from these public databases.

### Evolutionary Status of *H. pluvialis*

In total, 260 single-copy gene families including 3,640 genes were obtained from the 14 representative algal species. These genes from each species were concatenated together and constituted a super-length nucleotide data set to yield 1,147,546 aligned sites. Our phylogenetic analysis supports three main groups among the examined species, which is consistent with the putative classification of Prasinococcales, Trebouxiophyceae, and Chlamydomonadales. The *H. pluvialis* clade obviously located into the group of Chlamydomonadales and displayed a closer relationship with *V.carteri*, *C.reinhardtii*, and *M.neglectum* than other species. Thus, at the first time, we demonstrated the phylogenetic position of *H. pluvialis* at a whole-genome level. Interestingly, the *H. pluvialis* clade displays the longest branch length in Chlamydomonadales (supplementary fig. S1, Supplementary Material online). Since the branches are in units of substitutions per site with calculation by MrBayes (version 3.2.2; Ronquist et al. 2012), the result suggests that *H. pluvialis* has a faster nucleotide substitution rate during the evolution of flagellated green algae.

### Genomic Basis for the High Production of Astaxanthin in *H. pluvialis*

*Haematococcus pluvialis* is well known for its capacity to produce large amounts of astaxanthin, a strong antioxidant for aquaculture and cosmetics. Commercially, more than 40 g of astaxanthin can be extracted from 1 kg of dry cells (Lorenz and Cysewski 2000), leading this species to be an ideal resource for astaxanthin production. A previous study (Grunewald et al. 2001) reported that the putative pathway of astaxanthin biosynthesis (fig. 1*d*) consists of two key enzymes (CRTO and CRTR-B). Interestingly, CRTO is encoded by β-carotene ketolase gene (*bkt*). Three *bkt* genes were previously confirmed to be upregulated when the *H. pluvialis* cells were induced in different stress conditions (Huang et al. 2006). Therefore, the *bkt* genes were considered as one of the major contributors to the rapid accumulation of large amounts of astaxanthin.

In our current study, we employed the three reported BKT protein sequences (Huang et al. 2006) as queries to search our genome assembly. Interestingly, we identified six *bkt* copies in *H. pluvialis* genome with a wide distribution in different scaffolds. However, only one corresponding ortholog was identified in each of the phylogenetically closed species (*V. carteri*, *C. reinhardtii*, and *M. neglectum*). Among the six *bkt* copies in *H. pluvialis*, we determined that three copies are more similar to *bkt1*, thus named them as *bkt1a*, *bkt1b*, and *bkt1c* respectively; since two copies are similar to *bkt2*, we defined them as *bkt2a* and *bkt2b*; the rest one is *bkt3*. Through the phylogenetic analysis (fig. 1*e*), we observed that the six copies of *bkt* genes clustered into a single clade, which is separated from the single *bkt* ortholog from *V. carteri*, *C. reinhardtii*, and *M. neglectum*. In addition, these *bkt* copies in *H. pluvialis* can be divided into two groups, in which one consists of *bkt1*s, while the other contains *bkt2*s and *bkt3*. In fact, among the nucleotide sequences, *bkt2*s and *bkt3* are highly similar and display slight site changes in their protein sequence alignments; however, *bkt1* presents relatively more site changes, especially within the initial 220 bp (supplementary fig. S3, Supplementary Material online).

### Transcriptome Profiling and Validation of Novel Potential Genes for Astaxanthin Production

Isopentenyl pyrophosphate (IPP) is a key intermediate of carotenoid synthesis, and there are two independent pathways that produce IPP in algae cells, namely the mevalonate pathway (MVA) in the cytosol and nonmevalonate (MEP) in chloroplasts (Shah et al. 2016). Earlier investigations have shown that the MVA pathway has been lost in many green algae (Chlorophyta) and red algal taxa. Previously, only two MVA pathway enzymes (acetyl-CoA C-acetyltransferase [ACAT] and hydroxymethylglutaryl-CoA synthase [HMGS]) were identified in the *H. pluvialis* transcriptomes (Gwak et al. 2014). Here, by searching our genome and transcriptome data, we could only find these two genes for the MVA pathway too. The loss of hydroxymethylglutaryl-CoA reductase (HMGR), mevalonate kinase (MVK), phosphomevalonate kinase (PMK), and mevalonate diphosphate decarboxylase (MVD) in the genome of *H. pluvialis* confirms that as a member of the Chlorophyta, *H. pluvialis* exclusively uses the MEP pathway to synthesize IPP. This might be a common phenomenon in the green algae (Lohr et al. 2012; Qiu et al. 2016).

The molecular mechanisms underlying astaxanthin synthesis in *H. pluvialis* (Shah et al. 2016) and stress factors such as light intensity (He et al. 2018; Ma et al. 2018), salinity (Han et al. 2013), temperature (Hong et al. 2015), and chemical substances (Gao et al. 2015; Zhao et al. 2015) have been extensively studied. Here, we further examined the effects of high irradiation and salinity on the accumulation of astaxanthin in *H. pluvialis* and identified some novel genes that may participate in this process. By comparing with the control (LLMT) groups, we identified 1,121 DEGs (log_2_ [ratio] ≥1 and *P*_adj_ ≤0.05) in the treated (HLST) groups, with 482 up-regulated and 639 down-regulated (supplementary table S4, Supplementary Material online). Subsequently, the KEGG enrichment analysis clustered all these DEGs into 103 KEGG pathways, including carotenoid biosynthesis (ko00906), terpenoid backbone biosynthesis (ko00900), and biosynthesis of unsaturated fatty acids (ko01040) (supplementary table S5 and fig. S2, Supplementary Material online).

There were six DEGs enriched into the carotenoid biosynthesis pathway, including phytoene synthase (PSY), phytoene desaturase (PDS), and beta-carotene hydroxylase (CrtR-b). All these DEGs were up-regulated in the treated HLST groups (supplementary table S6, Supplementary Material online). Except for *CrtR-b*, the rate-limiting enzyme CRTO (Fraser et al. 1997, Choi et al. 2006) was also eliminated from the DEG list, with a slight elevation of 30–70%. The upregulation of all six genes in the pathway of astaxanthin biosynthesis is consistent with the findings of previous reports (Han et al. 2013; He et al. 2018), in which high light and salinity enhance astaxanthin production in *H. pluvialis.*

### Long-Read Reference Transcriptome of *H. pluvialis*

A total of 157,416 isoforms were identified after removing the chloroplast, mitochondrial, and ribosomal transcripts. The length of these sequences among this data set ranged from 50 bp to 14,995 bp, with a N50 of 5,106 bp. An isoform-level reference transcriptome set of 18,483 transcripts with high confidence was generated.

Alternative splicing and polyadenylation can contribute to the diversity of transcripts (Abdel-Ghany et al. 2016; Wang et al. 2016). We therefore employed AStalavista (Foissac and Sammeth 2007, 2015) to identify the five main modes of alternative splicing, including intron retention, exon skipping, alternative 3′ splice site, alternative 5′ splice site, and mutually exclusive exons. Interestingly, the final results revealed that the intron retention was the most abundant mode, while no mutually exclusive exon was identified in these *H. pluvialis* transcripts (supplementary table S7, Supplementary Material online).

## Conclusions

We report the first whole-genome sequencing, assembly, and annotation of the astaxanthin-producing green microalga, *H. pluvialis*. This draft genome assembly is a valuable genetic resource for elucidating the deep genetic basis for astaxanthin production. In our present study, we observed a remarkable expansion of the *bkt* gene family in the *H. pluvialis* genome, which may contribute to high astaxanthin yield. Transcriptome sequencing highlighted that several important pathways were involved in astaxanthin synthesis. These genomic and transcriptomic data may be utilized in elucidating the molecular mechanisms underlying astaxanthin yield and accumulation, which in turn will facilitate breeding of novel strains with significantly higher astaxanthin content.

## Supplementary Material

Supplementary data are available at *Genome Biology and Evolution* online.

## Author Contributions

Z.H. and Q.S. designed the project. C.B., Y.L., and Y.H. assembled and annotated the genome. Y.L. and C.B. performed the evolution analysis. Q.L., M.T., and Y.Z. collected the samples and prepared the quality control. Y.H., Q.L., and M.T performed the transcriptome analysis. M.T., Y.Z., C.W., J.L., B.J., J.c.L., Z.L., and J.X. participated in data analysis and figure preparation. C.B., M.T., Q.S., Q.L., Z.H., Y.L., and Y.H. prepared the manuscript. All authors read and approved the final manuscript.

## Supplementary Material

Supplementary Data
