# Supplementary material for: Genome and Transcriptome Sequencing of the Astaxanthin-Producing Green Microalga, Haematococcus pluvialis
Source: Genome Biol Evol. 2018 Nov 29;11(1):166–73. doi: 10.1093/gbe/evy263 (PMC6330051; doi:10.1093/gbe/evy263)
Supplement: Supplementary Data [file evy263_supp.zip › Supplementary Data revised.docx]

**Supplementary Tables**

**Supplementary Table 1.** RNA-seq evaluation of gene coverage in the genome assembly.

| Dataset | Number | Total length | Covered by assembly (%) | with >50% sequence in one scaffold | |
| --- | --- | --- | --- | --- | --- |
|  |  |  |  | **Number** | **Percentage (%)** |
|  |  |  |  |  |  |
| >200bp | 53405 | 41832424 | 92.8% | 51074 | 95.6 |
| >500bp | 21164 | 31805739 | 91.6% | 19709 | 93.1 |
| >1000bp | 12339 | 25621578 | 90.9% | 11291 | 91.5 |

**Supplementary Table 2.** Mapping data of each transcriptome sample to the generated genome assembly.

| **Sample Group** | **HLSTA** | **HLSTB** | **HLSTC** | **LLMT4** | **LLMT5** | **LLMT6** |
| --- | --- | --- | --- | --- | --- | --- |
| Total Clean Reads  Total Mapping %  Uniquely Mapping %  Total Gene No.  Known Gene No.  Novel Gene No.  Total Transcript No.  Known Transcript No.  Novel Transcript No. | 47984828  85.14%  42.93%  22513  16783  5730  42181  6919  35262 | 50248474  85.65%  42.86%  22243  16591  5652  41794  6696  35098 | 50392078  85.54%  42.30%  22403  16694  5709  42231  6952  35279 | 51614202  84.55%  42.25%  22574  16835  5739  42691  7178  35513 | 59057482  83.71%  41.70%  22609  16859  5750  43066  7356  35710 | 50665756  84.71%  42.66%  22585  16829  5756  42639  7123  35516 |
|  | | | | | | |

**Supplementary Table 3.** Gene transcription levels (FPKM values) in the six samples.

See separated excel file

**Supplementary Table 4.** List of the identified 1,121 differentially expressed genes (DEGs) with the KEGG annotation.

See separated excel file

**Supplementary Table 5.** List of the enriched 103 KEGG pathways from all the differentially expressed genes (both up- and down-regulated).

See separated excel file

**Supplementary Table 6.** List of genes involved in the astaxanthin biosynthesis and accumulation.

See separated excel file

**Supplementary Table 7.** Statistics of alternative splicing in *H. pluvialis*.

| **Classification** | **Intron retention** | **Exon skipping** | **Alternative 5’splice site** | **Alternative 3’splice site** | **Mutually exclusive exons** |
| --- | --- | --- | --- | --- | --- |
| **Number** | 337 | 27 | 46 | 35 | 0 |

**Supplementary Figures**

**
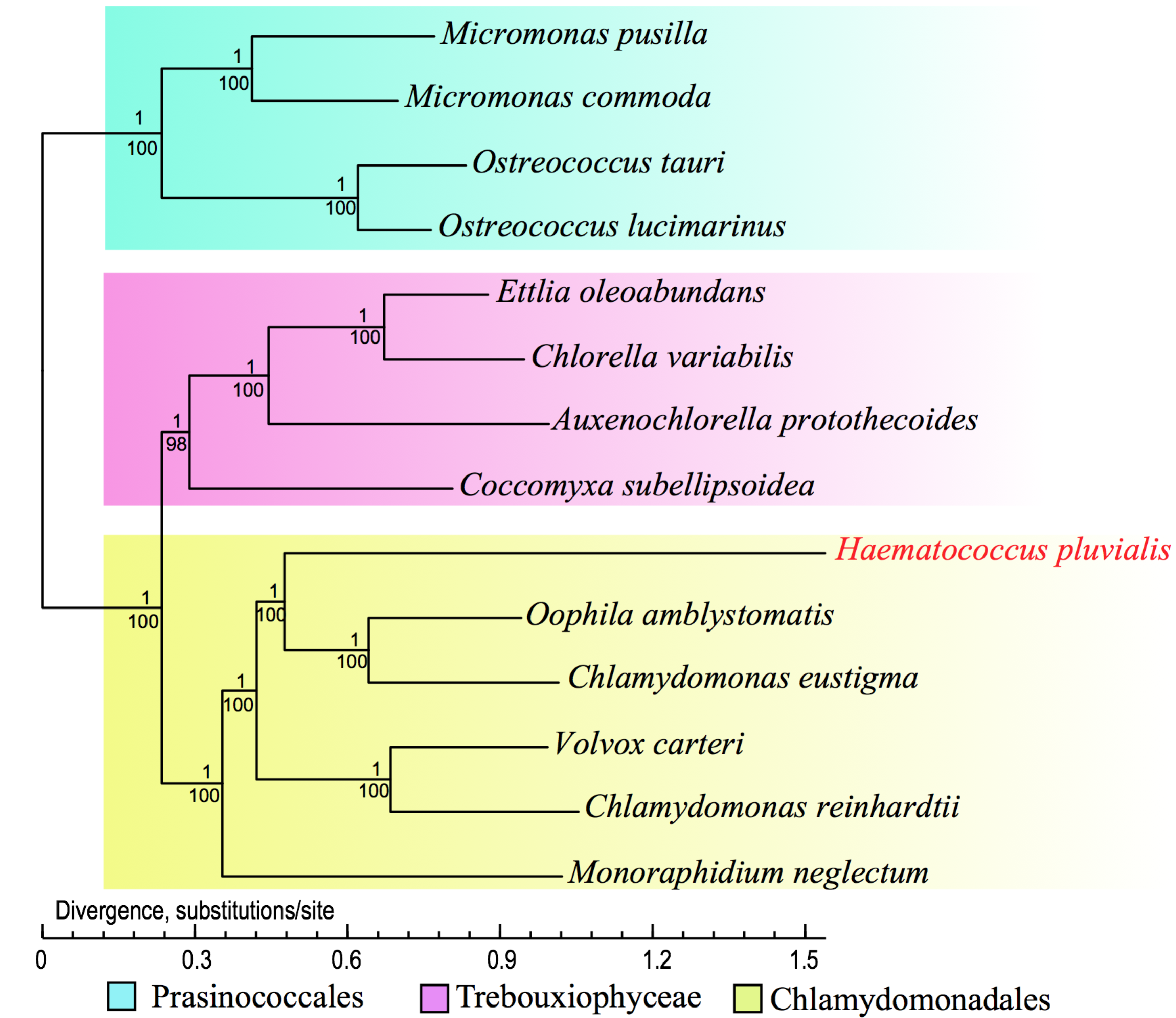
**

**Supplementary Figure 1. The evolutionary relationship of** ***H. pluvialis* and other 13 algae.** The robust phylogenetic topology suggested classification of three major groups. *H. pluvial is* clustered with the stem branch of *O. amblystomatis* and *C. eustigma*, which belonged to the group of Chlamydomonadales. Interestingly, *H. pluvialis* has the longest length branch among all the examined species, indicating its higher substitution rate than others.

**
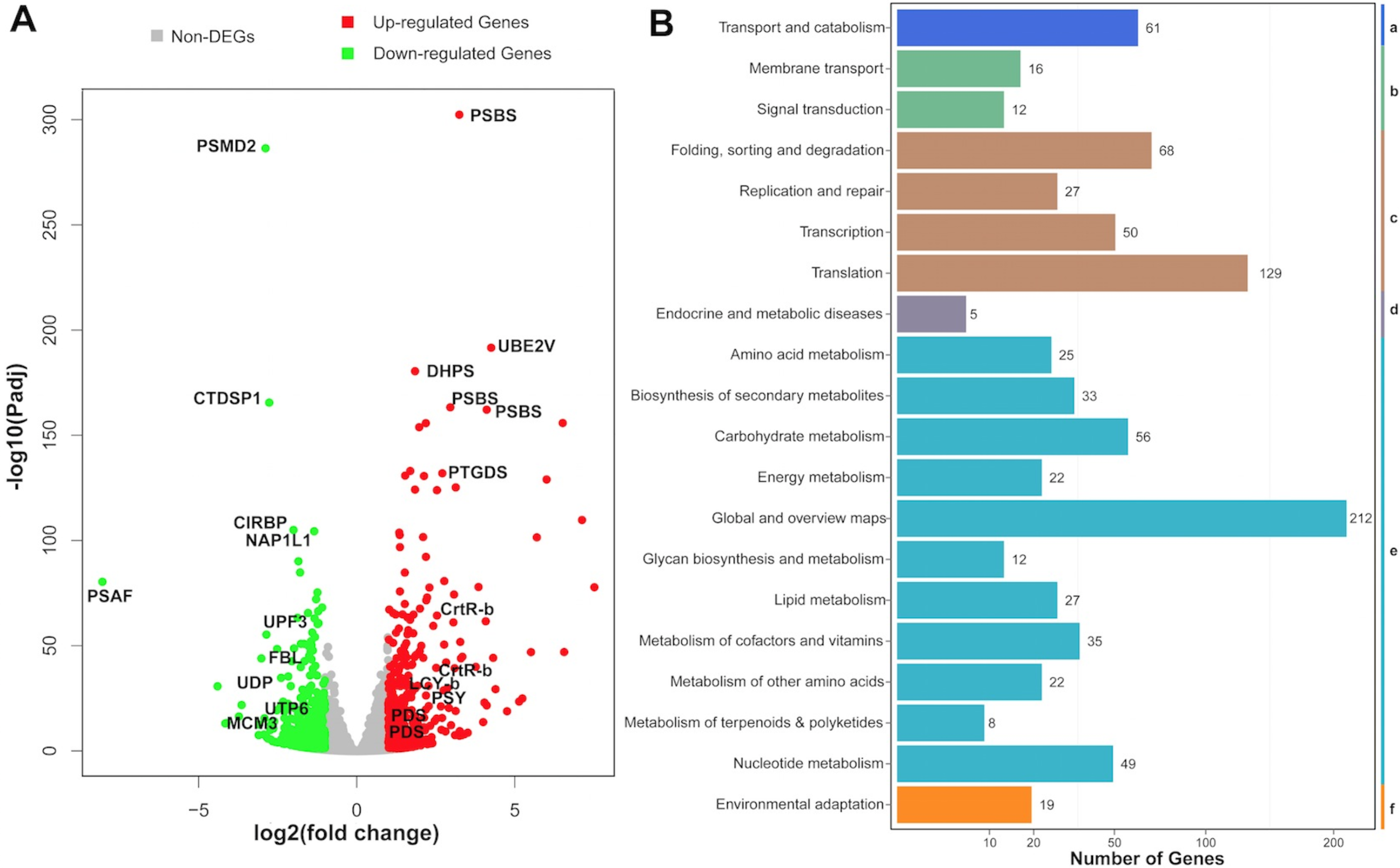
**

**Supplementary Figure 2. Differentially expressed genes between LLMT and HLST groups for the enrichment of KEGG pathways. A.** Volcano plot of the identified 1,121 DEGs. Each dot (no matter red, green or grey) represents a gene. Extremely differentially expressed genes and those related to astaxanthin biosynthesis were denoted with abbreviated names. **B.** All DEGs were enriched into 103 KEGG pathways that can be classified into 20 groups (level 2) and six classes (level 1; a: Cellular Processes, b: Environmental Information Processing, c: Genetic Information Processing, d: Human Diseases, e: Metabolism, and f: Organismal Systems).


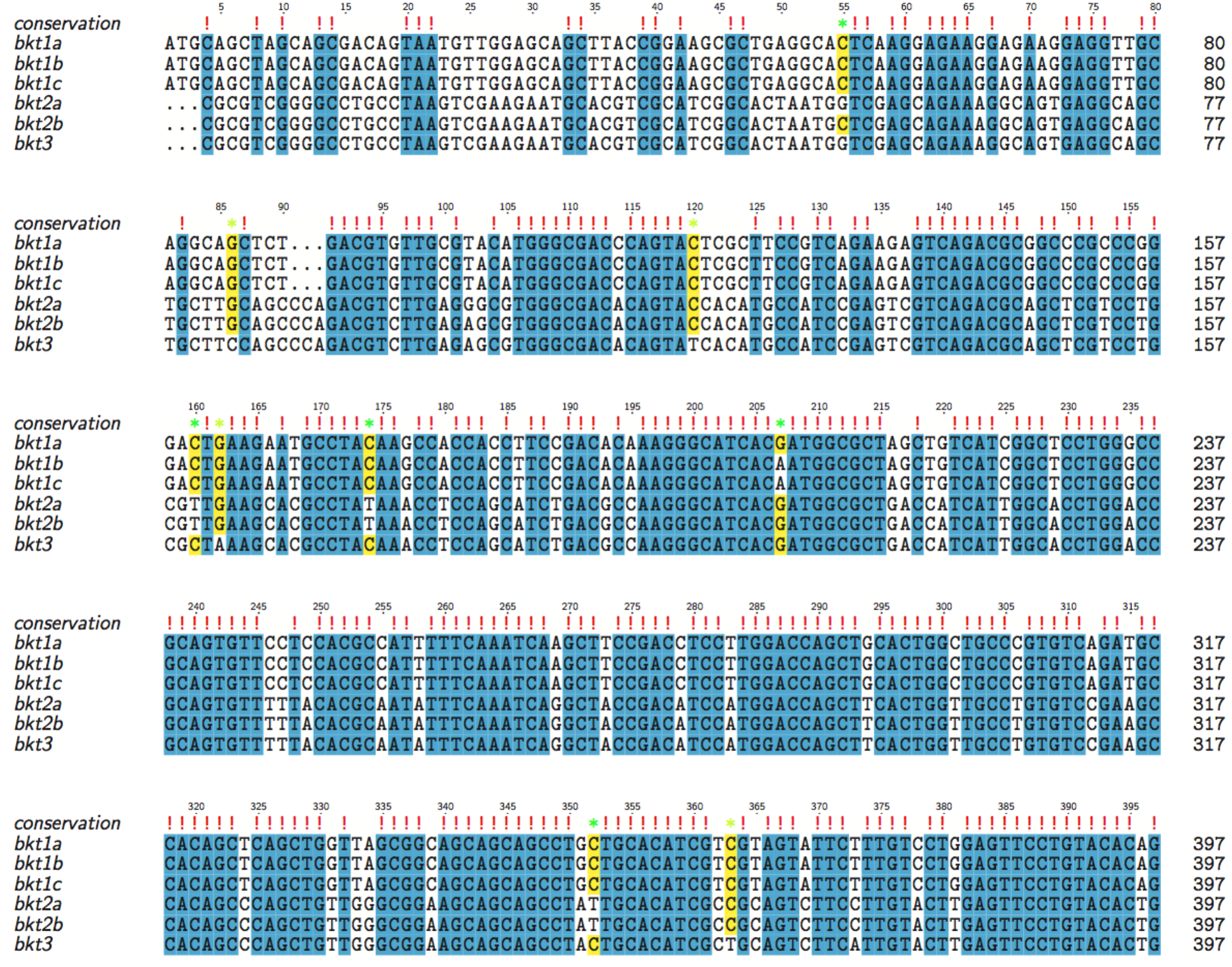

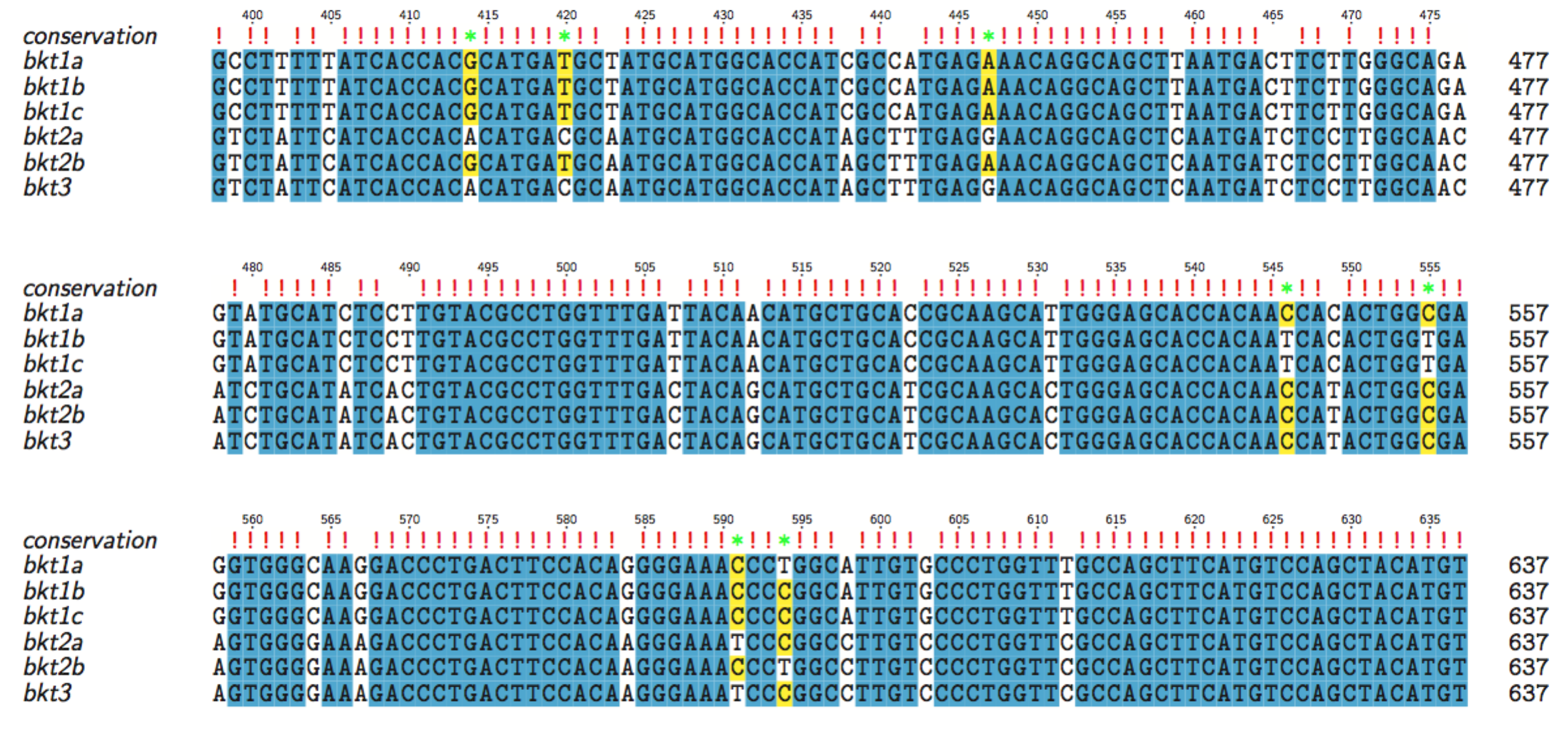


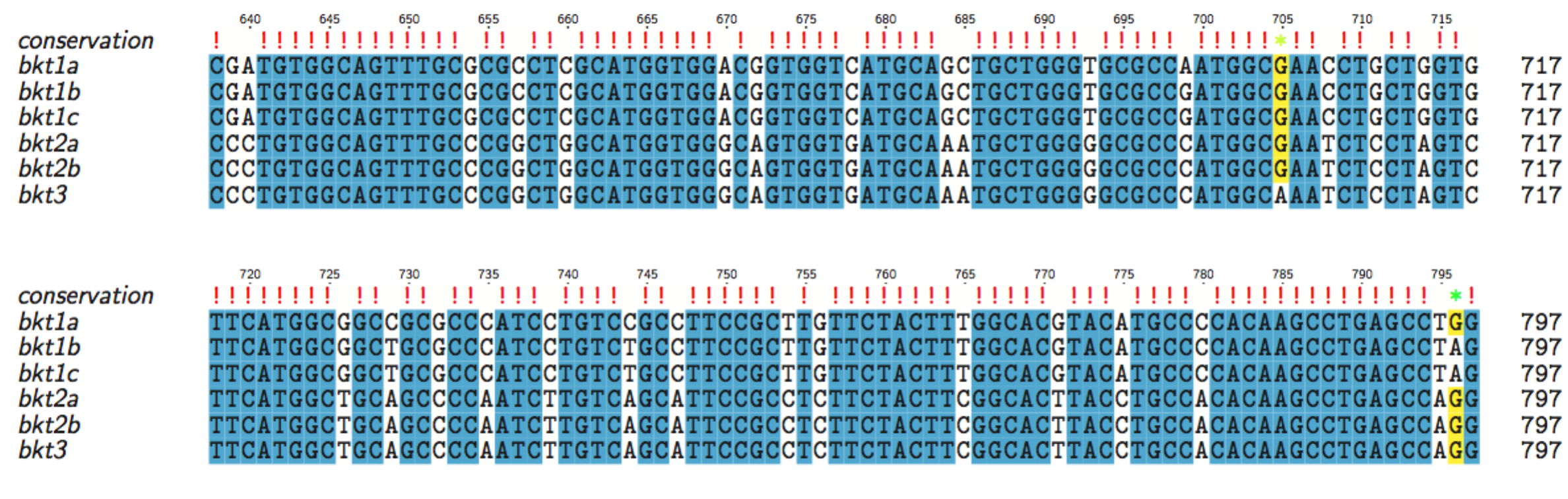


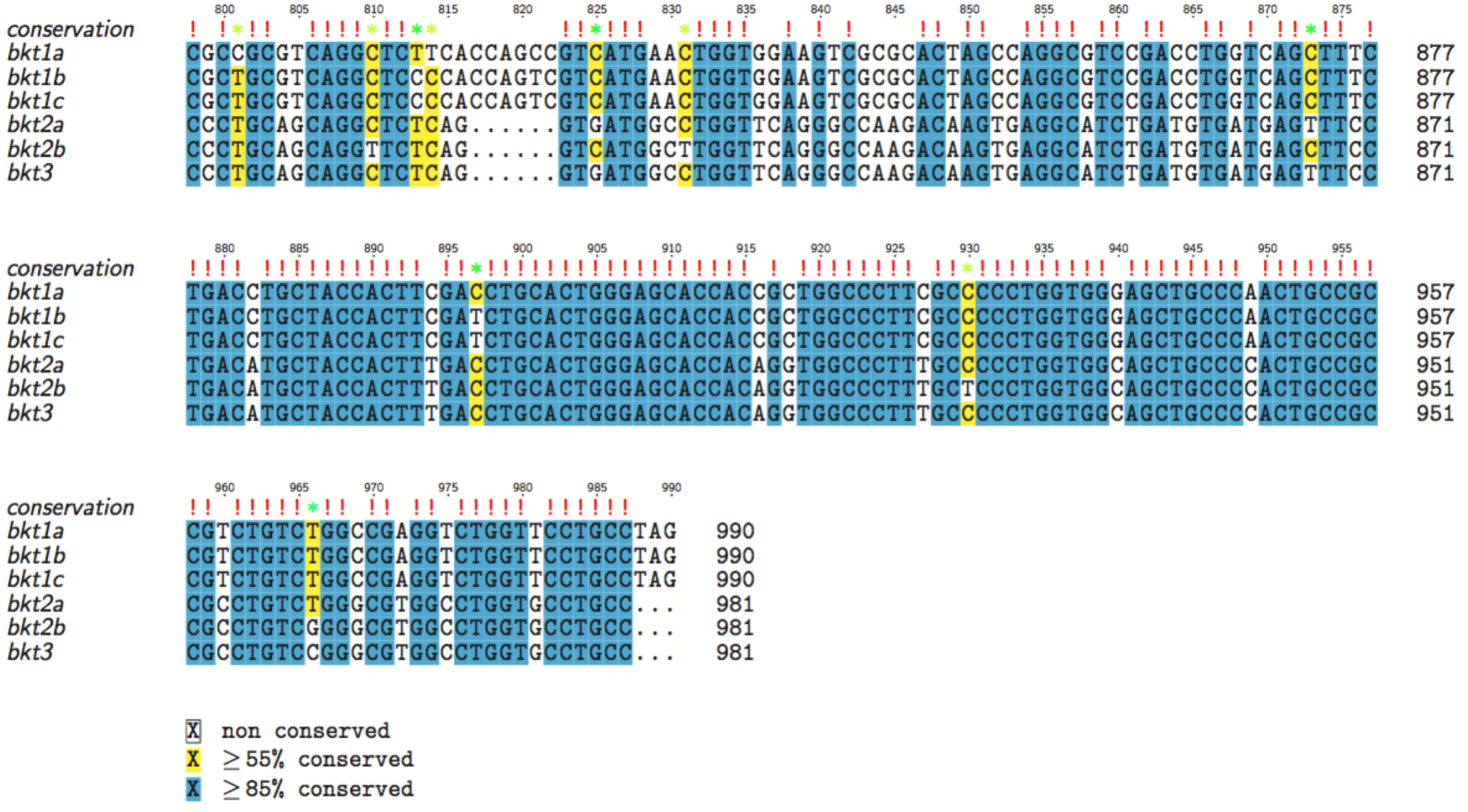


**Supplementary Figure 3. Alignment of protein sequences for the six *bkt* genes in *H. pluvialis*.**
