# Supplementary material for: Genome and Transcriptome Sequencing of the Astaxanthin-Producing Green Microalga, Haematococcus pluvialis
Source: Genome Biol Evol. 2018 Nov 29;11(1):166–73. doi: 10.1093/gbe/evy263 (PMC6330051; doi:10.1093/gbe/evy263)
Supplement: Supplementary Data [file evy263_supp.zip › Supplementary legends.docx]

**Supplementary Table 1.** RNA-seq evaluation of gene coverage in the genome assembly

**Supplementary Table 2.** Mapping data of each transcriptome sample to the generated genome assembly

**Supplementary Table 3.** Gene transcription levels (FPKM values) in the six samples

**Supplementary Table 4.** List of the identified 1,121 differentially expressed genes (DEGs) with the KEGG annotation

**Supplementary Table 5.** List of the enriched 103 KEGG pathways from all the differentially expressed genes (both up- and down-regulated)

**Supplementary Table 6.** List of genes involved in the biosynthesis and accumulation of astaxanthin

**Supplementary Table 7.** Statistics of alternative splicing in *Haematococcus pluvialis*

**Supplementary Figure 1. The evolutionary relationship of** ***H. pluvialis* and other 13 algae.** The robust phylogenetic topology suggested classification of three major groups. *H. pluvial is* clustered with the stem branch of *O. amblystomatis* and *C. eustigma*, which belonged to the group of Chlamydomonadales. Interestingly, *H. pluvialis* has the longest length branch among all the examined species, indicating its higher substitution rate than others.

**Supplementary Figure 2. Differentially expressed genes between LLMT and HLST groups for the enrichment of KEGG pathways. A.** Volcano plot of the identified 1,121 DEGs. Each dot (no matter red, green or grey) represents a gene. Extremely differentially expressed genes and those related to astaxanthin biosynthesis were denoted with abbreviated names. **B.** All DEGs were enriched into 103 KEGG pathways that can be classified into 20 groups (level 2) and six classes (level 1; a: Cellular Processes, b: Environmental Information Processing, c: Genetic Information Processing, d: Human Diseases, e: Metabolism, and f: Organismal Systems).

**Supplementary Figure 3. Alignment of protein sequences for the six *bkt* genes in *H. pluvialis*.**
